# Magnetic Field Stabilization for Magnetically Shielded Volumes by External Field Coils

**DOI:** 10.6028/jres.110.020

**Published:** 2005-06-01

**Authors:** T. Brys, S. Czekaj, M. Daum, P. Fierlinger, D. George, R. Henneck, Z. Hochman, M. Kasprzak, K. Kohlik, K. Kirch, M. Kuzniak, G. Kuehne, A. Pichlmaier, A. Siodmok, A. Szelc, L. Tanner

**Affiliations:** Paul-Scherrer-Institut, CH-5232 Villigen, Switzerland

**Keywords:** Helmholtz coils, magnetic field stabilization, magnetic noise

## Abstract

For highly sensitive magnetic measurements, e.g., a measurement of the neutron electric dipole moment (EDM), the magnetic field has to be stable in time on a level below picoTesla. One of several measures we employ to achieve this uses an external field coil system which can stabilize the ambient external field at a predefined value. Here we report on the construction and characterization of such a system in the magnetic test facility at PSI. The system actively stabilizes the field along the axis of the EDM experiment by means of four coils in a Helmholtz-like configuration. Additional coils serve to compensate for transverse ambient field components. Because of the long integration times in the EDM experiment (about 100 s or more) only slow disturbances have to be corrected for. The performance of the system has been measured using static and moving magnetic sources and suppression factors in excess of 200 have been observed.

## 1. Introduction

The influence of magnetic fields on sensitive equipment can be reduced by up to several orders of magnitude by passive shielding with soft iron alloys (e.g., Mu-metal, Permalloy, Conetic). The suppression factor obtained in this way depends on the geometry of the shielding system, the shielding material properties and the characteristics of the field. For large volumes and unfavourable geometries—as they may be dictated by other requirements of the experiment—passive shielding may not be sufficient and other means of field stabilization or field noise reduction have to be applied. For instance, in the new experiment to measure the neutron electric dipole moment (EDM) in preparation at PSI the magnetic field has to be stable in time on a level below pT. This has to be compared to the amplitude of the natural and man-made variations of the magnetic field which can be as large as 100 nT in our case. Since most of these disturbances vary on a timescale of minutes to hours and the EDM experiment is essentially insensitive to moderate disturbances on a timescale below about 10 s we decided to test an external Helmholtz-like coil system which would be able to cancel slow ambient field variations. The basic idea is simple and similar to that of normal Helmholtz stabilization systems: a pickup sensor measures the ambient field value near the sensitive device, from which an error signal is derived with respect to a selectable set value. The error signal is converted into a current signal which is then fed to the external coil system surrounding the device. If the device has its own magnetic shielding, then the question arises whether the pickup sensor should be located within the passive shielding or outside. For good passive shielding, e.g. using at least 3 to 4 layers, the suppression factor due to the passive shielding alone is usually better than 1000 and the field level inside the shielding can get into the regime of several tens of pT. At this level the momentary field value measured by the pickup sensor may become influenced by ‘shielding’ noise which has nothing to do with changes of the ambient field outside. Thus there is no constant relationship between an outside disturbance and the corresponding correction signal and the stabilization will eventually break down.

Several such stabilization systems have been described in the literature, either in combination with soft-iron shielding [1–3], or with aluminum shielding [4], suppressing only ac magnetic fields. With soft-iron shielding of only one [2] or two [3] layers the pickup sensors were located within the shielding and stabilization suppression factors *k* as high as 400 have been observed [3]. However, it should be mentioned that the periodic magnetic disturbance used to obtain these results was produced by a double-coil system with almost identical field geometry as that of the compensation coil system. Under these conditions *k* is close to the limit imposed by the stabilization electronics system and is no measure for the suppression due to a realistic disturbance at large distances, i.e. ≥ 30 m. The suppression of what is stated as ‘real’ environmental noise is mentioned in Ref. [2] but not quantified. When the pickup sensor was located outside the shielding the suppression was observed to be much more modest, i.e. *k* ≤ 2 at frequencies around 100 mHz [3]. Reference [1] investigated a combined soft-iron/aluminum shielding with 3 layers and the pickup sensor outside the shielding. The cubic shielding was complemented by a Helmholtz-like external coil pair to compensate for vertical field components. The response to ‘vertical disturbances’ produced by a test solenoid at 15 m distance, but close to the symmetry plane of the external coil pair, yielded suppression factors up to 50 at 0.1 Hz.

## 2. Setup

**Fig. 1:**
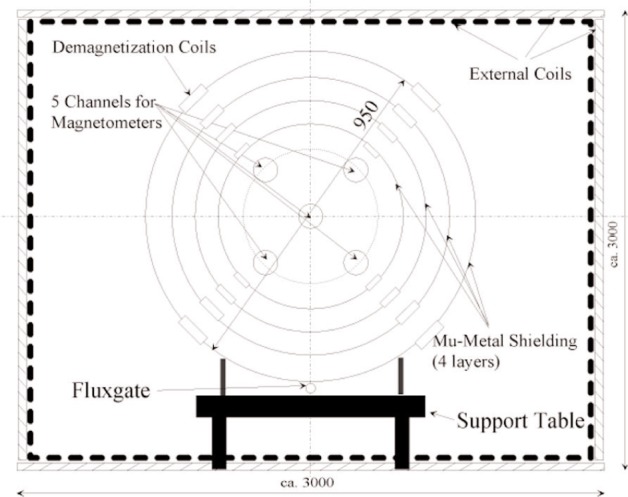
Principle of the experiment. The common axis of the 4 shielding cylinders is along *z* (with *z* pointing out of the paper plane). For details see text.

**Fig. 2:**
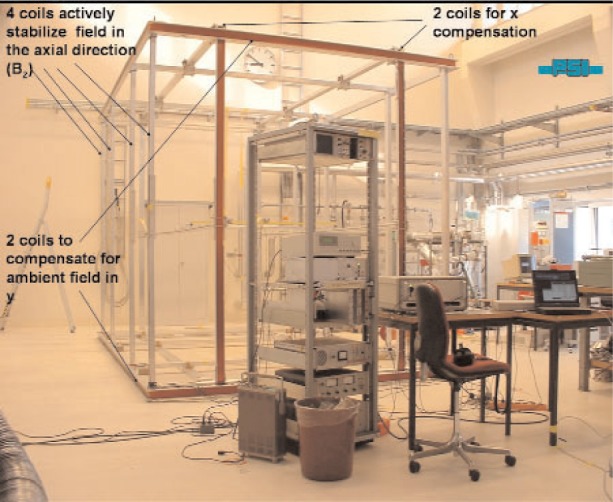
Photo of the external coil system. For details see text.

The principle of the experiment is sketched in Fig. 1 and consists of the permalloy shielding (79 HM), the external coil system and the sensor systems with the stabilization electronics. Figure 2 shows a photo of the external coil system. The common axis of the 4 shielding cylinders (with endcaps) is along *z* (pointing out of the plane in Fig. 1, +*y* pointing vertically down) and coincides with the axis of the EDM experiment. Its centre was defined as the origin of the coordinate system. The axial suppression factor of the shielding is predicted to be on the order of 10^3^, while the transversal one should be by about a factor of 10 higher. Therefore the main requirements for the external coil system were to

1. Provide zero or near-zero field in the axial direction (*B_z_*) and actively stabilize this component on the basis of feedback sensors. The bandwidth of the stabilization should be in the range of about 0.001 to 1 Hz in order to eliminate slow to medium-fast variations due to natural changes of the Earth field, slow truck movements, crane and elevator movements, magnets etc.
2. Compensate for the static ambient field and provide zero or near-zero field in transverse directions (*B_x,y_*). This is required to optimally demagnetize the outermost permalloy shield.

Given these requirements the stabilized field volume should be over the full permalloy shielding (1.6 m long and 0.95 m diameter for the outermost layer and 1.3 m, 0.61 m diameter for the innermost layer). The external coil system consists of:

- two rectangular coils for each transverse direction (3 m by 4.6 m) in a Helmholtz-like configuration at a spacing of 1.8 m (3m) for the *x* (*y*) direction. These two coil systems are powered in constant-current mode.
- 4 rectangular coils for the axial direction (3 m by 3 m) at 1.8 m and 4.2 m spacing to provide near-homogeneous field compensation over the 1.6 m length of the shielding. The 4 coils are connected in series and are powered by the stabilization power supply which is controlled by a correction signal derived from one (or two) fluxgates.
- 2 independent test coil systems (one for the axial direction, one for *x*) were mounted directly on the main coils. These were used to stimulate artificial field variations in order to measure the ideal suppression factor as a function of frequency. They could be powered by a special power supply which was stimulated by a signal generator to produce a sinusoidal current excitation with adjustable amplitude and frequency.

The digital regulation system uses the fluxgate input signals (up to two signals can be used for stabilization, with individually selectable weighting factors) which are sampled at about 50 kHz and filtered with a simple low-pass filter at about 240 Hz. After digitization the values are compared to a selectable set-value and the correction values thus produced are sent to the power converter with maximum amplitude 7 A/20 V. The control system consists of two nested control PI loops, the parameters of which were specified by empirical trials. The sensors used are 3-axial fluxgates (Bartington Mag-03MC).

The electronic performance of the stabilization was tested with a sinusoidal stimulation of the test coils (which have the same geometry as the Helmholtz coils) and using one fluxgate in the centre of the apparatus as feedback sensor. This was done before installation of the permalloy shielding. The suppression factor *k_z_* = *B_z_*^stab off^/*B_z_*^stab on^ was determined from the peak-to-peak amplitudes of *B_z_* with and without stabilization. Given the close-to-ideal spatial matching between test coil and compensation coil one expects large suppression factors, limited essentially only by the stabilization electronics. Indeed, the measured *k_z_* values ranged from ∼10 at 50 Hz to ∼10^4^ at 1 mHz. The frequency response of the stabilization was optimized for frequencies ≤ 1 Hz.

Next, we included the permalloy shielding and the Cs-magnetometer system. The axial shielding factor of the shielding (developed and provided by PNPI Gatchina, St. Petersburg) was found to be about 100. This is a factor 20 below expectation, however consistent with low measured susceptibilities and is attributed to improper tempering of the fully welded shielding cylinders. Good demagnetization can be achieved with an induction transformer, delivering a smoothly decreasing demagnetization current. The Cs-magnetometer system was developed at Vavilov State Optical Institute, St. Petersburg [5] and Ioffe Physical Technical Institute, St. Petersburg. Cs-magnetometers belong to the class of atomic magnetometers which are based on detection of Larmor spin precession of optically pumped atoms, approaching sensitivities close to several fT · Hz^−1/2^ for large measurement volumes. The Cs quantum magnetometers used here operate in self-oscillating mode with gas-discharge driven Cs-lamps. The small magnetic field (*B_z_* ≈ 2 µT) required to operate the magnetometers (and the EDM experiment in future) was provided by a solenoid mounted inside the shielding and driven by a highly stable current source. Any transverse, external field component transmitted through the shielding is thus negligible with respect to this solenoid field and the magnetometers—which are in principle scalar devices—measure essentially only the *B_z_* component. 5 magnetometers were available which could be moved along the *z*-direction in the 5 channels indicated in Fig. 1.

The external coil system is enclosed in a thermal housing which, in combination with a temperature stabilization system, provides stable temperatures on a level below 0.1 K.

## 3. Measurements and Results

**Fig. 3:**
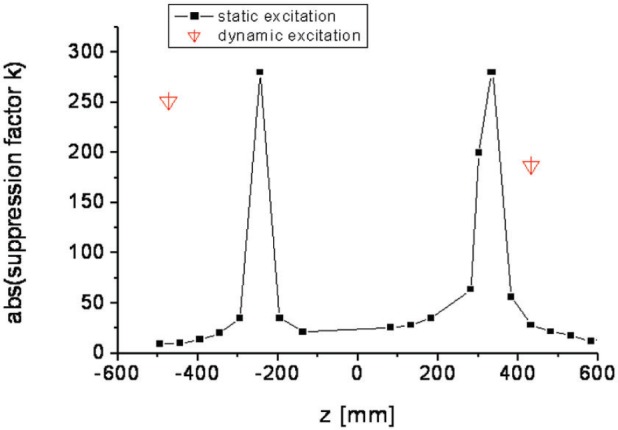
Absolute value of the stabilization suppression factor |*k_z_*| as a function of the pickup sensor position, shown here for the case *x* = 0, *y* = −50 cm, *z* variable.

For testing we employed stationary as well as moving magnetic sources. In the first case we used a small solenoid positioned at *x* = −7 m, *y* ≈ 50 cm, *z* ≈ 0 and its axis parallel to *z*. It produced *B_z_* amplitudes ≤ 100 nT at the center of the device, i.e. at *x* = *y* = *z* = 0 (with the shielding removed, of course). The solenoid was simply used in on/off mode with signal duration of several seconds. For moving sources we employed various types of vehicles which were driven along the road parallel to *z* at about 11 m distance. This is a situation identical as for the future site of the EDM experiment. The measurement procedure was as follows: the response of the Cs magnetometers within the shielding to the external magnetic source was measured, with and without application of the stabilization system and the suppression factor *k_z_* calculated as defined above. The pickup sensor position was varied, but always as close as possible to the shielding (i.e. with minimum 1 cm spacing), below and above the shielding, i.e. at *x* = 0, *y* = ±50 cm, *z* variable. Results are given in Fig. 3 where |*k_z_*| is plotted vs. the pickup sensor position, for the stationary and the dynamic case (where for clarity we have only displayed the two points with maximum suppression). For both types of test sources the sensor location was found to be very sensitive, such that a shift of a few cm destroyed the good performance with |*k_z_*| ≥ 200. For the static case over-compensation (*k_z_* < 0) was observed for *z*-values between the two maxima and under-compensation (*k_z_* > 0) elsewhere. It should be noted that our stationary test geometry is similar to the one reported by Ref. [1], who reported suppression factors up to 50 at 0.1 Hz. Qualitatively, this appears to be consistent with our optimum values which were obtained at zero frequency limit. In the dynamic case each datum corresponds to an average over 10 drive-by’s with stabilization at roughly the same speed normalized to an average over 10 drive-by’s without stabilization at roughly the same speed.

**Fig. 4:**
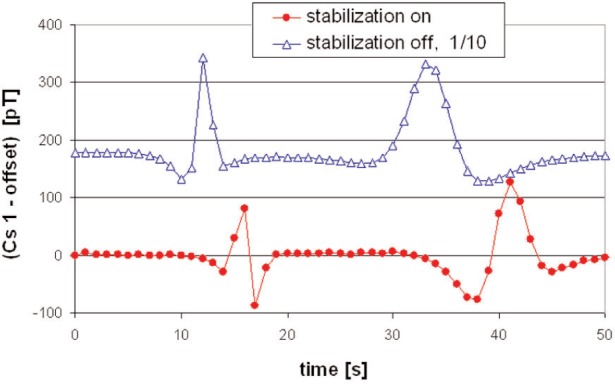
Cs magnetometer response to a moving source (a VW bus driving parallel to *z* at about 11 m distance): without stabilization (open triangles), with stabilization (filled circles). Please note that suitable offsets have been subtracted for convenient display and the data without stabilization have been divided by a factor 10. The first signal in time corresponds to the vehicle driven at a speed of about 25 km/h, the second one to a speed of about 10 km/h.

The response to a dynamic source is shown in Fig. 4. It is seen that the suppression effect derives not only from a reduction of the signal amplitude but also from the change of the signal shape. The essentially unipolar signal without stabilization becomes transformed into a bipolar signal with stabilization and since in an EDM experiment one integrates over times on the order of 100 s the integrated signal with stabilization becomes very small and *k_z_* correspondingly large. The uncertainty for *k_z_* is estimated to be on the order ≤30 % for large *k_z_* for the following reasons: (a) in the stationary case the signal level (≈5 pT) for large *k_z_* is close to the noise level; (b) in the dynamic case the data taking rate for the Cs magnetometers is fixed to 1 Hz and given the time structure of the signal with stabilization large uncertainties must be expected after integration due to such a coarse time binning.

The linearity of *k_z_*(stationary) was measured at small *k_z_* with lower excitation amplitudes and found to be valid over the range 10 to 100 nT. In the dynamic case we also used other types of vehicles producing smaller disturbances (“normal” cars, fork-lifters) with essentially the same results. We also tested whether *k_z_* depends on the Cs-magnetometer position within the shielding and found that it is essentially homogenous over the tested position range |*z*| ≤ 50 cm.

While for the dynamic case the test scenario corresponds perfectly to reality, the stationary test case used is specific for the position of the test solenoid and applies only for a disturbance signal arising at a fixed position (a similar case was tested in Ref.[1] and yielded k ≤ 50 at 0.1 Hz). This conclusion was tested by keeping the pickup sensor position fixed and moving the test solenoid parallel to *z* at *x* ≈ 4 m. Large *k_z_* was observed only for a very narrow range of positions (−10 cm ≤ *z* ≤ +10 cm) around *z* ≈ 0. Outside this optimum range *k_z_* quickly dropped and remained more or less constant at *k_z_* ≈ 5.

**Fig. 5:**
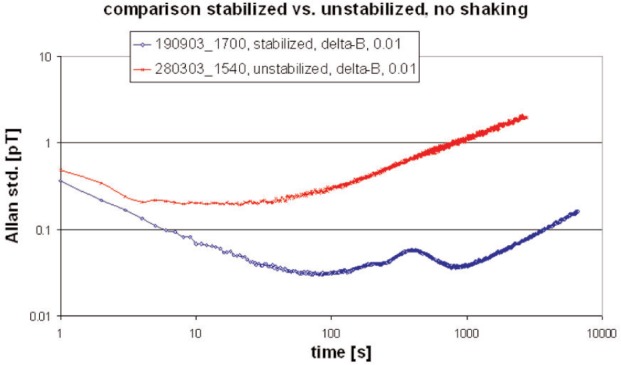
Allan standard deviation for the quantity Δ*B*(*t*) = *B_c_*(*t*) − (Σ*B_i_*(*t*))/4 and the stabilization system switched on (lower curve) or off (upper curve). For further detail see text.

The response to the ambient, unperturbed field noise was investigated using the concept of the Allan standard deviation [6] which represents a convenient noise measure as a function of averaging (or integration) time. In order to eliminate spatially homogeneous field fluctuations we used the results from all 5 magnetometers and calculated the quantity Δ*B*(*t*) = *B_c_*(*t*) − (Σ*B_i_*(*t*))/4, where *B_c_*(*t*) is the reading of the magnetometer at *x* = *y* = *z* = 0 and the *B_i_*(*t*), *i* = 1,..4 are the readings of the 4 magnetometers in the outer channels at *z* = 0 (see Fig. 1). The Allan standard deviation of this quantity is plotted in Fig. 5 as a function of averaging time with and without stabilization system switched on. Measurements were done overnight or on week-ends with the thermal stabilization turned on to avoid “thermo-currents” in the shielding. From Fig. 5 it is obvious that within the time window interesting for an EDM experiment, i.e. between 100 to 1000 s, the stabilized system performs better by at least one order of magnitude. Near the minimum values around 30 fT are obtained which is sufficient for the EDM experiment. It should be noted, however, that the value at minimum reaches 1 pT if the 4 outer magnetometers are positioned at the extreme ends of the shielded volume, i.e. with essentially 1 m spacing.

We close by adding that similar performance as with the external coil stabilization was obtained by using “shaking”, i.e. permanent demagnetization with an ac current of at least 1 A, applied to the two outer shielding layers. Using shaking together with the stabilization surprisingly did not lead to an improvement. It appears that for the given shielding the noise limit observed cannot be improved further by “external” means and that the shielding itself imposes the limit. Possible mechanisms could be connected to magnetic composition, history, interaction between shield and stabilization field and between shield and the internal solenoid field. To test for the latter mechanism we performed a cross-comparison of the Cs-magnetometers (which require the 2 µT internal field) with a LTS-SQUID magnetometer (without internal field), provided by PTB Berlin. The results [7] demonstrate that there is indeed some internal field noise contribution, but only for averaging times below 10 s while above 10 s no difference was observed.

## 4. Conclusions

A 3-dimensional external stabilization system in Helmholtz-like configuration was built and tested in conjunction with a 4-layer permalloy shield and the pickup sensor outside the shield. Measurements in dc mode with a stationary test solenoid yielded suppression factors ≥200 for certain positions of the pickup sensor. This however only applies for well defined placement of the test solenoid. Signals from moving vehicles on a nearby road parallel to the system axis are suppressed by a factor of about 200, again strongly dependent on the pickup sensor location. Finally, real ambient noise was found to be suppressed by a factor 10 to 20 in the frequency range 1 to 10 mHz.
